# E-Cigarette Awareness, Use, and Harm Perception among Adults: A Meta-Analysis of Observational Studies

**DOI:** 10.1371/journal.pone.0165938

**Published:** 2016-11-18

**Authors:** Ying Xu, Yanfang Guo, Kaiqian Liu, Zheng Liu, Xiaobo Wang

**Affiliations:** 1 Department of Chronic non-communicable disease prevention and control, Baoan Chronic Diseases Prevent and Cure Hospital, Shenzhen, China; 2 Department of Nutrition, School of Public Health, Guangdong Pharmaceutical University, Guangzhou, China; Dartmouth College Geisel School of Medicine, UNITED STATES

## Abstract

**Objective:**

The aim of this study is to systematically review the published literature on the awareness, previous and current use, and harm perceptions of electronic cigarettes (e-cigarettes) among adults.

**Methods:**

A search of the most current literature using the PubMed and Scopus database to identify articles published since 2003 yielded a total of 28 relevant articles.

**Results:**

The pooled prevalence of awareness, previous use, current use of e-cigarettes and perceived healthier of e-cigarettes than regular cigarettes (healthier perception) among adults were 61.2% (95% confidence interval (CI): 51.5–70.8%), 16.8% (95% CI: 14.0–19.6%), 11.1% (95% CI: 9.2–13.1%), and 52.6% (95% CI: 42.5–62.6%), respectively, using a random effects model. The subgroup analysis showed that pooled estimates were highest in the group of current smokers of regular cigarettes, except that the highest pooled rate of current use was seen in the group of former smokers of regular cigarettes (the corresponding rates were 71.9% (95% CI: 57.5–86.3%), 27.2% (95% CI: 18.8–35.6%), 16.8% (95% CI: 7.2–26.3%), and 63.1% (95% CI: 52.1–74.1%)), and the lowest pooled rates were in the group of non-smokers, except for the rate of healthier perception in the users of e-cigarettes (and the corresponding rates were 46.8% (95% CI: 26.8–66.8%), 2.5% (95% CI: 1.1–5.6%), 1.2% (95% CI: 0.4–2.1%), and 37.9% (95% CI: -0.5–76.3%)). The cumulative meta-analysis found that awareness increased over time, while the prevalence of previous use, current use, and healthier perception first experienced an increase followed by a decrease and remained stable thereafter.

**Conclusions:**

E-cigarette awareness has been increasing, and e-cigarette use and perceived health risks are nearly invariable between 2009 and 2014. Given the substantial heterogeneity in the prevalence rate estimates, there is a need for more accurate and comparable prevalence estimates for e-cigarettes across the world.

## Introduction

The electronic cigarette (e-cigarette), first developed in China in 2003, is a battery-powered nicotine delivery device that provides inhaled doses of vaporized nicotine solution and is designed to look and feel like a traditional cigarette. E-cigarettes have become increasingly popular, for example, it was reported that e-cigarettes sales have doubled every year in the United States (US) since 2008[1]. This increasing trend might be due to e-cigarette product promotional campaigns conducted on multiple mainstream marketing channels, including television, print, radio, and the Internet. Kornfield et al. reported that promotional spending in the US was minimal through mid-2010 and has since rapidly increased, reaching US$12 million in 2011 and US$22 million in 2012. For the second quarter of 2013 alone, expenditures reached US$28 million, over eight times more than spending in the second quarter of 2012[2]. Luo et al. systematically assessed 196 unique videos and found that 94% (n = 185) were “pro” e-cigarettes and 4% (n = 8) were neutral, while only 2% (n = 3) were “anti” e-cigarettes[1].

However, there is insufficient scientific evidence concerning the health benefits of e-cigarettes, possible adverse effects, the efficiency of cigarette cessation attempts and cigarette abstinence[3–6]. Consequently, approaches to regulation vary widely. Some states have labeled e-cigarettes as tobacco products, while others have passed measures that define them as something else, such as “alternative nicotine products” or “vapor products” [7]. In a revised European Union Tobacco Product Directive, e-cigarettes have been regulated as tobacco products or medical devices, depending on the nicotine concentrations (up to 20 mg/ml or more than that or not [8]). Some countries, such as Brazil and France, have banned the sale, import, and advertising of e-cigarettes, while other countries such as Finland have treated e-cigarettes as medicinal products and banned only their advertising [9]. In addition, some local governments in the US have taken action to prohibit sales to minors or otherwise restrict e-cigarettes use. The World Health Organization (WHO) has called for regulations that impede the promotion of e-cigarettes, minimize their potential health risks, and prohibit unproven health claims [10].

To face this new challenge, more and more scientific research has been conducted to identify the safety and efficacy of e-cigarettes worldwide. A bibliometric analysis [11] indicated that the number of published articles in the PubMed database on this topic has grown exponentially, from less than 100 in 2012 to more than 200 in 2013 to more than 800 in 2014. WHO is currently reviewing the existing evidence on e-cigarettes to understand their impact on health (Statement revised on 3 June 2014 in GENEVA). This study aimed to review the increasing published literature related to e-cigarettes awareness, use, and perceived harmfulness among adults and to combine these results using meta-analysis. In this case, evidence-based decision-making might inform efforts to determine appropriate public health policy and regulatory action.

## Methods

### Search strategy

We searched the electronic databases PubMed (2003 to Feb 2015) and Scopus (2003 to Feb 2015) using the following broad set of search terms for article titles: “electronic cigarette OR electronic cigarettes OR e-cigarette OR e-cigarettes OR electronic nicotine delivery.” Additional articles were identified by checking the reference sections of relevant articles and previous systematic reviews of e-cigarettes.

### Study selection

Two reviewers (Xu and Guo) independently evaluated the eligibility of the studies identified by our search parameters according to the predetermined criteria and procedure. Disagreements between the reviewers were resolved through discussion until a consensus was reached.

The inclusion criteria we used were: 1) the study sample included either young adults or adults; 2) one or more of the following four rates were reported: e-cigarette awareness (have previously heard of e-cigarettes), previous use (have tried an e-cigarette in their lifetime), current use (have tried an e-cigarette in their lifetime and used it in the last 30 days), and perception about the safety of e-cigarettes (believing that e-cigarettes are healthier than regular cigarettes, i.e,, healthier perceptions); 3) sample size was reported or 95% CI of the rate was estimated; and 4) an observational study, including a cross-sectional study and a cohort study, was performed. The exclusion criteria were: 1) the study sample included only adolescents; 2) none of the four above-mentioned rates was estimated; 3) the studies were clinical trials, intervention studies, quality research (including focus groups), or a case report; 4) the study had a sample size of less than 200 (given the reliability of the estimated rate of awareness, use, and healthier perception of e-cigarettes). Non-English articles were also excluded.

**Fig 1:**
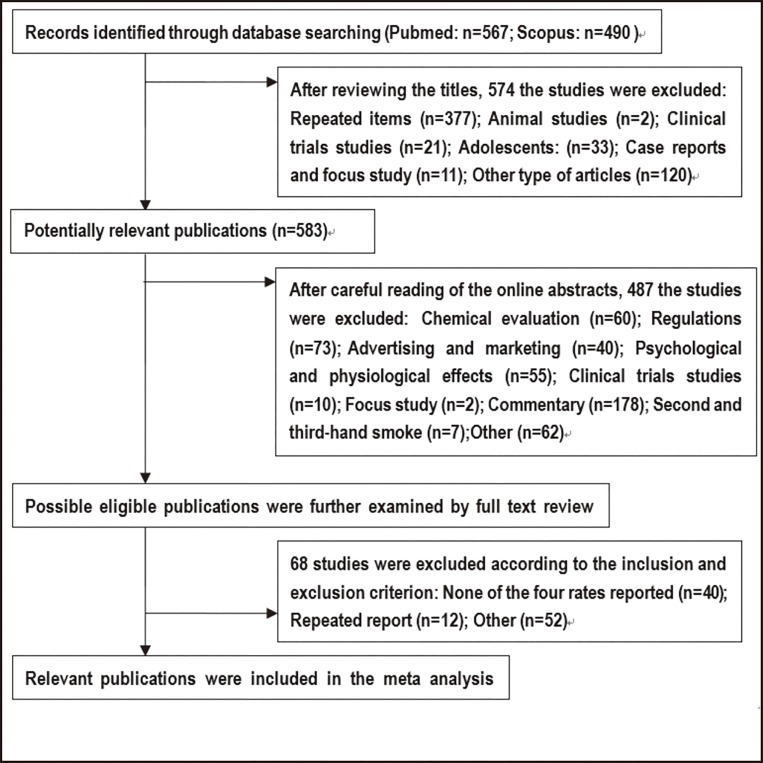
Flow chart of literature search and article identification.

The procedure of identifying articles was as follows (Fig 1): 1) the repeated items and obviously irrelevant studies were excluded after reading the titles; next 2) article abstracts were screened for potential relevance; and then 3) the full texts were reviewed. Using this procedure, 28 articles were deemed relevant for this analysis according to the inclusion and exclusion criteria.

### Data extraction and quality evaluation

After careful reading of these articles, 28 articles were included, and the appropriate Strengthening the Reporting of Observational Studies in Epidemiology (STROBE) checklist [12] was used to the quality of each article. The STROBE checklist of 22 items relate to the title, abstract, introduction, methods, results, and discussion sections of articles. Every checklist item was evaluated. Articles that met at least eight of the STROBE criteria were accepted for meta-analysis.

For each study, the following information was extracted: first author’s last name; year of publication; country or area of survey; survey time period; age of subjects; smoking status; number of subjects; and the prevalence of awareness, previous use, current use, and healthier perception of e-cigarettes; and the corresponding 95% CIs. In addition, if there were results obtained from two or more surveys using similar survey methods in the same article, the most recent one would be extracted and included in the meta-analysis. All the data for the analysis were available in S1 File.

### Statistical analysis

Stata Statistical Software (version 11; Stata Corporation, College Station, TX, US) was used to analyze the data. First, the standard error of the prevalence of e-cigarettes awareness, use, and healthier perception was calculated for each study using the binomial distribution formula. Statistical heterogeneity was then assessed using the I^2^ statistic and χ^2^ test. If statistical heterogeneity was evident across the studies (p-value of χ^2^ test < 0.05 and I^2^ > 50%), a random-effects model proposed by DerSimonian and Laird was selected to estimate the overall prevalence and the 95% confidence intervals (CIs). Otherwise, a fixed-effect model was applied. Third, a sensitivity analysis was conducted by omitting one study at a time to investigate the influence of a single study on the overall estimate. Finally, Egger’s test and Begg’s test were employed to assess publication bias. In addition, analysis was conducted for each smoking status subgroup. In this study, seven subgroups were defined according to smoking status and available information from the published articles: 1) Current smokers (defined as smoking at least 100 cigarettes in his or her lifetime and endorsed current “every day” or “someday” smoking); 2) Former/ever smokers (defined as having smoked at least 100 cigarettes in his or her lifetime and endorsed no current smoking); 3) Never smokers (defined as having smoked less than 100 cigarettes in his or her lifetime); 4) Current and ever smokers (defined as 1) and 2)); 5) Smokers and non-smokers (defined as all participants without considering his or her smoking status); 6) Users of e-cigarette (defined as having used e-cigarettes in the last 30 days) and 7) Awareness of e-cigarette (defined as having ever heard of e-cigarettes).Cumulative meta-analysis was also performed to summarize changes to the overall estimates with time by adding one study at a time in the order of survey time.

## Results

### Study characteristics

A total of 28 studies satisfied all the inclusion and exclusion criteria (Fig 1). The majority of the included studies (n = 16) were carried out in the US; three in the United Kingdom; one each in Poland, the Czech Republic, New Zealand, Italy, Canada, and Spain; one in four countries (including the US, the UK, Canada, and Australia); one in a number of areas (including Europe, the US, and Canada); and one in 33 countries (72% in Europe). The survey time ranged from 2009 to 2014, including 15 surveys in 2010–2011, 8 in 2012 and 5 in 2013. The survey subjects included current smokers, former/ever smokers, and non-smokers. Notably, most of the data were collected from online surveys except for the three surveys in Spain, Italy, and the Czech Republic, which were collected through face-to-face interviews and one from a sample of a cohort study in four states (MN, SD, ND, and MI) in the US. All but one of the included studies met more than 18 criteria from the STROBE checklist of 22 items, one study met 8 criteria, as shown in Table 1.

**Table 1:** Description of the individual studies included in the meta-analysis.

Id	1^st^ Author	Year	Country	Period of study	Population	Age (Years)	Sample size	Awareness of e-cig	Previous use of e-cig	Current use of e-cig	Healthier perception of e-cig	STROBE score
1	Adkison[19]	2013	U.S.,UK,CA,AU*	2010.7–2011.6	Current and former smokers	>18	5939	0.466	0.076	0.029	0.798(current smokers)	18
2	Choi[25]	2013	U.S.(MN,SD,ND,MI,KS)	2010.10–2011.3	Smokers and non-smokers	20–28	2624	0.699	0.07	0.012	0.53(awareness)	20
					smokers		532	0.85	0.285		0.593	
					Former smokers		320	0.745	0.097		0.525	
					Never smokers		1742	0.655	0.027		0.519	
3	Dawking[20]	2013	33 countries (72% in Europe)	2011.9–2012.5	Males,END users	43 (M)^#^	1347				0.06	20
4	Dockrell[26]	2013	Britain	2012.2	Smokers	>18	2093	0.79	0.216	0.03		21
					Former smokers		4473	0.38	<0.01	<0.01		
					Never smokers		5866	0.47	0.04	0.01		
				2010.4	Smokers		1380		0.35		0.39	
5	Etter[18]	2011	European union and U.S., CA	2010.3–10	Smokers and non-smokers, END users	41 (M)	3587		0.85	0.808	0.84(users)	20
					Smokers		1051		0.705	0.617	0.811(n = 740)	
					Former smokers		2508		0.91	0.892	0.843(n = 2279)	
6	Goniewicz[27]	2012	Poland	2010.9–2011.6	Smokers and non-smokers	15–24	13787	0.864	0.209	0.069	0.548	21
					Smokers		4738	0.431	0.437	0.153		
					Non-smokers		9022	0.087	0.087	0.024		
					Smokers and non-smokers	20–24	1894		0.19	0.059		
7	King[28]	2013	U.S.	2011.7–8	Smokers and non-smokers	>18	4050	0.579(0.558–0.600)	0.062(0.052–0.073)			18
					Smokers			0.769(0.722–0.815)	0.212(0.170–0.254)			
					Former smokers			0.654(0.617–0.691)	0.074(0.050–0.097))			
					Never smokers			0.501(0.473–0.529)	0.013(0.007–0.018)			
8	Kralikova[29]	2012	Czech	2011.10	Smokers	32 (M)	973	0.86	0.26	0.07		8(Letter)
9	Li[30]	2013	New zealand	2011.3–5	Current and former smokers	>18	480	0.07			0.33	20
10	McMillen[31]	2012	U.S.	2010.9–11	Smokers and non-smokers	>18	3240		0.018	0.003546		21
					Smokers		580		0.067241379			
					Former smokers		787		0.015247776			
					Never smokers		1802		0.003329634			
11	Pearson[32]	2012	U.S.	2010.6	Smokers and non-smokers	>18	2649	0.402	0.03	0.01		21
					Smokers		1308	0.571	0.114(0.093–0.140)	0.041(0.03–0.056)	0.432	
					Former smokers		661	0.415	0.02(0.010–0.038)	0.0049 (0.0013–0.018)	0.46	
					Never smokers		680	0.325	0.77(0.0035–0.017)	0.0029 (0.001–0.008)		
				2010.1–4	Current and ever smokers	18–49	3658	0.58	0.06			
					Smokers		419	0.582	0.064(0.053–0.077)		0.46	
					Formersmokers		3239	0.58	0.031(0.013–0.071)		0.449	
12	Popova[33]	2013	U.S.	2011.11	Current and former smokers	42 (M)	1836		0.201	0.08		20
13	Regan[34]	2013	U.S.	2010.4–5	Smokers and non-smokers	>18	10328	0.322	0.027	0.01		19
					Smokers			0.496(0.459–0.533)	0.182(0.138–0.227)	0.063(0.041–0.086)		
					Former smokers			0.307(0.281–0.334)	0.062(0.040–0.083)	0.029(0.014–0.045)		
					Never smokers			0.283(0.262–0.303)	0.038(0.027–0.049)	0.022(0.013–0.031)		
14	Richaedson[35]	2012	U.S.	2010.6	Current and former smokers	>18	1310		0.1		0.35	18
15	Sutfin[36]	2013	U.S.(NC)	2009.fall	Smokers and non-smokers	21 (M)	4444		0.049	0.02	0.45(users)	20
					Smokers		867		0.106			
					Former smokers		882		0.074			
					Never smokers		2253		0.012			
16	Trummbo[37]	2013	U.S.(CO)	2011.4	Smokers and non-smokers	19–22	244	0.71	0.13			19
17	Vickerman[22]	2013	U.S.(CT,LA,NE,NC,SC,TX)	2012.1–10	Smokers	49 (M)	2476		0.309	0.09	0.045(users)	20
18	Pokhrel[38]	2014	U.S.(Hawaii)	?	Smokers	45.8 (M)	834		0.134			19
19	Tan[39]	2014	U.S.	2013.10–12	Smokers and non-smokers	49.5 (M)	1449		0.136			21
					Smokers		219		0.472			
					Former smokers		422		0.127			
					Never smokers		809		0.038			
20	Zhu[40]	2013	U.S.	2012.2–3	Smokers and non-smokers	>18	10041	0.754	0.081	0.014	0.499(users)	19
					Smokers		3111		0.322	0.063		
					Smokers			0.881(0.859–0.903)				
					Never smokers			0.692(0.670–0.714)				
21	Giovenco[41]	2014	U.S.	2013.6	Current and former smokers	>18	2136		0.468	0.161		18
					Former smokers				0.383(0.257–0.510)	0.139(0.047–0.231)		
22	Christensen[42]	2014	U.S.(Kansas)	2012–2013	Current and former smokers	>18	9656		0.118	0.034		20
					Smokers		1341		0.45			
					Former smokers		2593		0.105			
					Never smokers		5690		0.022			
23	Tan[43]	2014	U.S.	2012.10–2013.2	Smokers and non-smokers	>18	3630	0.771(0.745–0.797)n = 3487			0.507(0.478–0.537)(awareness, n = 2609)	21
					Smokers		586	0.886(0.844–0.928)			0.65(0.576–0.724)	
					Formersmokers		939	0.783(0.743–0.823)			0.495(0.436–0.553)	
					Never smokers		2052	0.730(0.686–0.773)			0.459(0.421–0.496)	
24	Gallus[44]	2014	Italy	2013	Smokers and non-smokers	>15	3000	0.911	0.068	0.012		20
					smokers				0.204	0.037		
					Former smokers				0.07			
					Never smokers			0.89	0.026			
25	Czoli[45]	2014	Canada	2012	Smokers and non-smokers	16–30	1188	0.434	0.161	0.057		20
26	Brown[46]	2014	Britain	2012	current and former smokers		4117	0.93	0.366	0.215	0.670	20
					Smokers		3538	0.934	0.365	0.219	0.676	
					Former smokers		579	0.929	0.373	0.188	0.632	
27	Vardavas[47]	2014	European union	2012	Smokers	>15	7352		0.203			20
					Former smokers	>15	5782		0.047			
					Never smokers	>15	13432		0.012			
28	Jose[48]	2014	Spain	2013–2014	Smokers and non-smokers	>16	736		0.065	0.016		21
					smokers		171		0.211	0.053		
					Former smokers		267		0.041	0.007		
					Never smokers		298		0.003	0.003		

Note

*U.S.,UK,CA,AU: the United states, the united kingdom,Canada and Australia; #(M):mean age.

### Awareness of e-cigarettes

**Fig 2:**
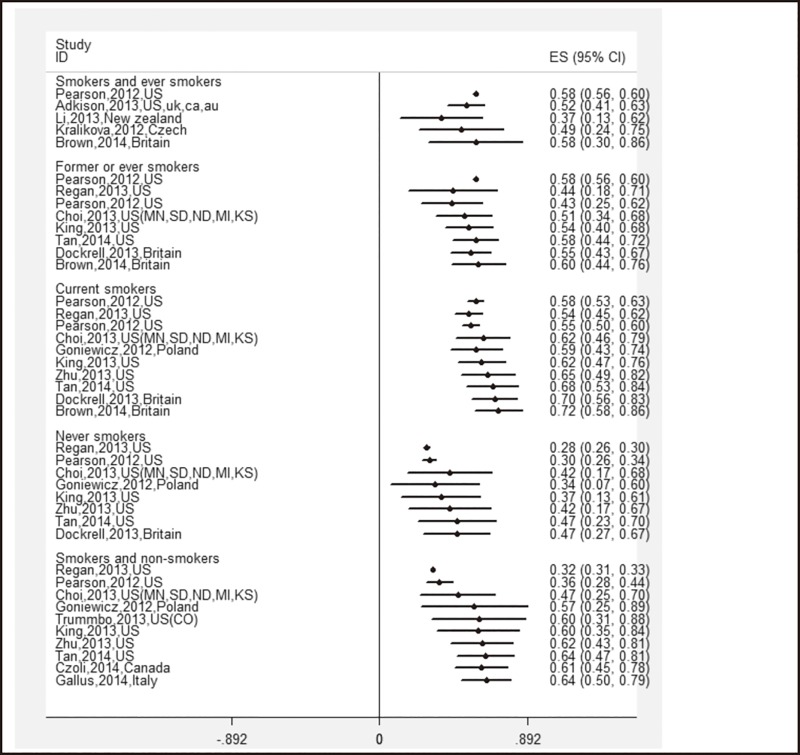
Cumulative meta-analysis of 41 studies’ e-cigarettes awareness by subgroups of different smoking status.

A total of 41 survey results were included in the meta-analysis of e-cigarette awareness, and the overall estimate was 61.2% (95% CI: 51.5%–70.8%). The subgroup analysis showed that the pooled estimates ranged from 46.8% (95% CI: 26.8%–66.8%) to 71.9% (95% CI: 57.5%–86.3%). The highest prevalence was among current smokers, and the lowest was among non-smokers (Table 2). The cumulative meta-analysis showed a tendency of growth across all subgroups since mid- 2010 (Fig 2).

**Table 2:** Pooled estimates for awareness, previous use, current use and healthier perception of e-cigarette using D+L random effect models.

	Awareness of e-cig		Previous use of e-cig		Current use of e-cig		Healthier perception of e-cig	
	N	Pooled estimate(95%CI)	N	Pooled estimate(95%CI)	N	Pooled estimate(95%CI)	N	Pooled estimate(95%CI)
Subgroups								
Current smokers	10	**0.719(0.575,0.863)**	15	**0.272(0.188,0.356)**	7	0.168(0.072,0.263)	7	**0.631(0.521,0.741)**
Former/ever smokers	8	0.599(0.437,0.761)	15	0.157(0.012,0.272)	7	**0.182(0.077,0.287)**	6	0.568(0.384,0.751)
Never smokers	8	**0.468(0.268,0.668)**	12	**0.025(0.011,0.036)**	5	**0.012(0.004,0.021)**	2	0.491(0.432,0.550)
Current and ever smokers	5	0.581(0.299,0.864)	9	0.247(0.153,0.341)	6	0.107(0.047,0.168)	3	0.464(0.235,0.693)
Smokers and non-smokers	10	0.645(0.497,0.792)	16	0.150(0.081,0.219)	13	0.086(0.051,0.122)	1	0.548(0.540,0.556)
Users of e-cigarette							5	**0.379(-0.005,0.763)**
Awareness of e-cigarette							2	0.520(0.498,0.543)
Overall	41	0.612(0.515,0.708)	67	0.168(0.140,0.196)	38	0.111(0.092,0.131)	26	0.526(0.425,0.626)

### Previous and current use of e-cigarettes

**Fig 3:**
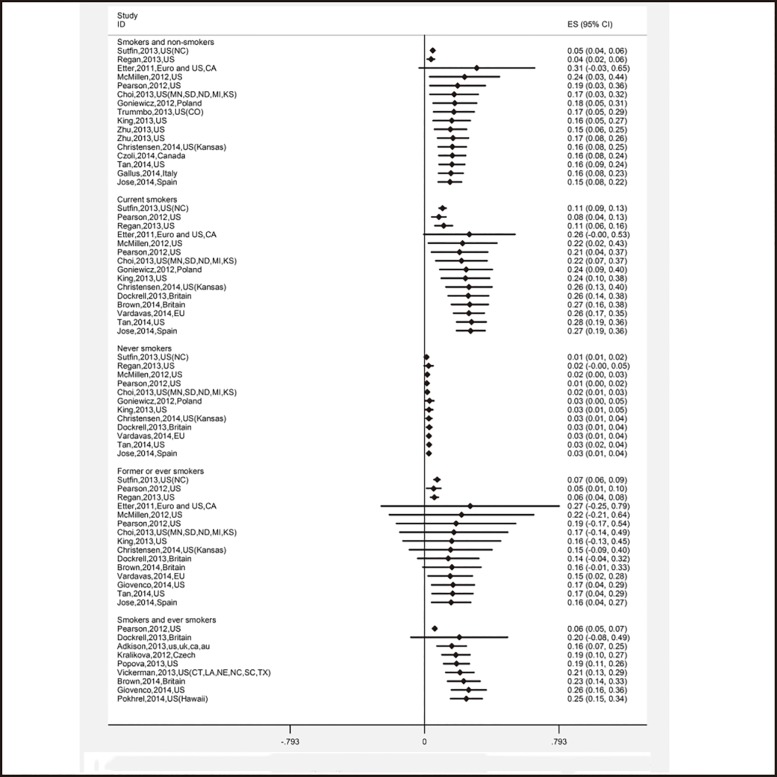
Cumulative meta-analysis of 67 studies’ ever use of e-cigarettes by subgroups of different smoking status.

A total of 67 survey results were included in the meta-analysis of the previous use of e-cigarettes. The overall estimate was 16.8% (95% CI: 14.0%–19.6%). Current smokers were more likely to try to smoke e-cigarettes than former smokers and non-smokers: 27.2% (95% CI: 18.8%–35.6%) vs. 15.7% (95% CI: 4.2%–27.2%) vs. 2.5% (95% CI: 1.4%–5.6%) (Table 2). The cumulative meta-analysis showed that the trends remained stable after first an increase and then a decrease among the three groups of current smokers, former smokers, and non-smokers (Fig 3).

**Fig 4:**
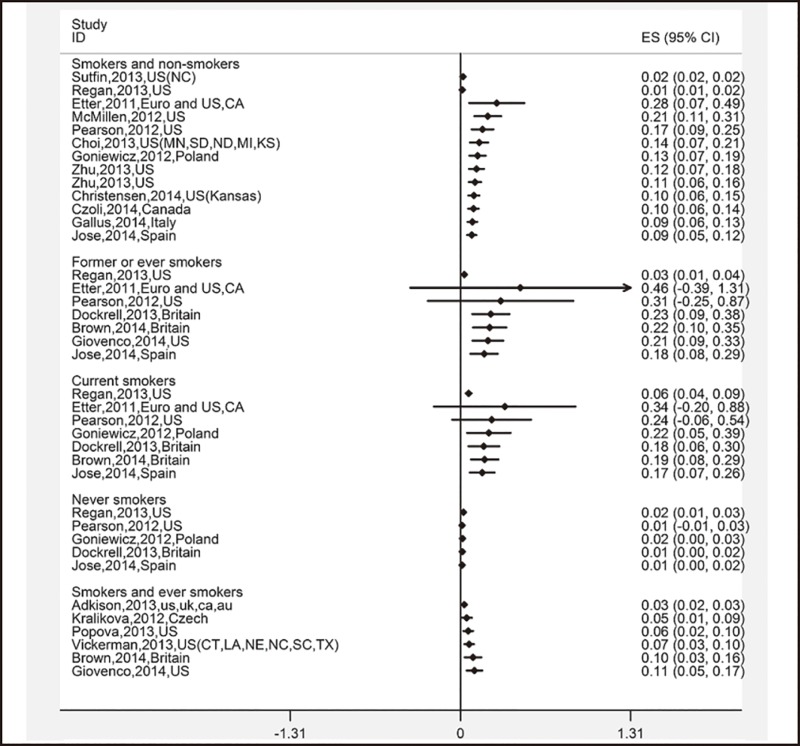
Cumulative meta-analysis of 38 studies’ current use of e-cigarettes by subgroups of different smoking status.

To analyze the current use of e-cigarettes, a total of 38 survey results were included. The pooled estimate was 11.1% (95% CI: 9.2%–13.1%). It was observed that 18.2% (95% CI: 7.7%–28.7%) of former smokers used e-cigarettes within the past 30 days, followed by current smokers (16.8% (95% CI: 7.2%–26.3%). There were 1.2% (95% CI: 0.4%–2.1%) of non-smokers who reported having used e-cigarettes in the past 30 days (Table 2). Additionally, the trends of current use of e-cigarettes observed in the cumulative meta-analysis were similar to the ones of previous use of e-cigarettes (Fig 4).

### Healthier perception of e-cigarettes

**Fig 5:**
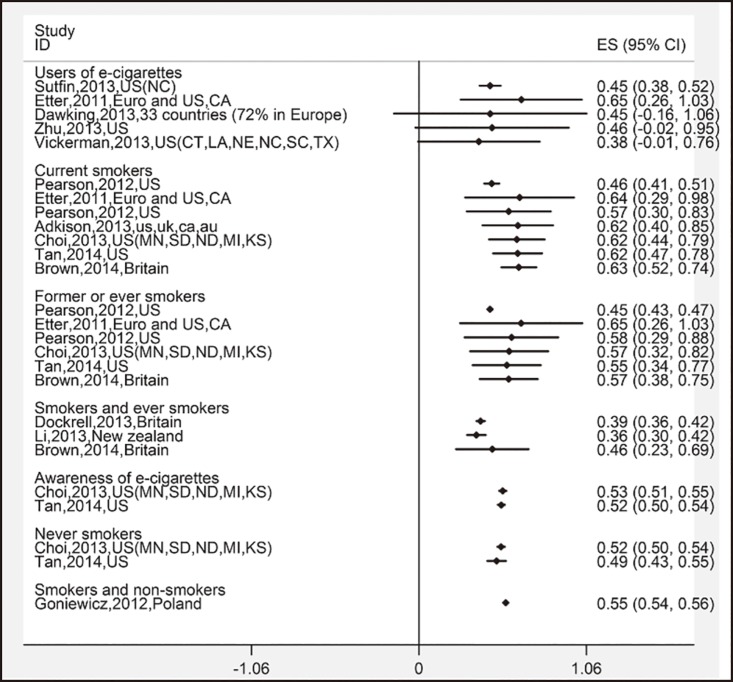
Cumulative meta-analysis of 26 studies’ harm perception of e-cigarettes by subgroups of different smoking status.

A total of 26 survey results were included in the meta-analysis. The overall estimate was 52.6% (95% CI: 42.5%–62.6%). Among users of e-cigarettes, only 37.9% (95% CI: -0.5%–76.3%) reported that they believed that e-cigarettes were healthier than conventional tobacco, and the proportion was lower than the ones for current smokers, former smokers, and non-smokers (Table 2). Trends of remaining unchangeable after first an increase and then a decrease were observed among the three groups of e-cigarettes users, current smokers, and former smokers in the cumulative meta-analysis, while the trend among non-smokers was not obvious because of limited studies (Fig 5).

### Sensitivity analysis, publication bias, and heterogeneity

Sensitivity analysis was performed to examine the robustness of the estimates. Each study was removed one at a time, and the meta-analysis was conducted repeatedly. Most of the estimates appear to be robust. However, the pooled prevalence of current users of e-cigarettes in the subgroup of former smokers changed from 18.2% (95% CI: 7.7%–28.7%) to 3.0% (95% CI: 1.8%–4.2%) when omitting the study conducted by Etter et al. in 2011 in Europe and CA, US which reported a high prevalence of current e-cigarette use (89.2%). Similar estimates were obtained before and after the removal of each study in both the total population and subgroups, indicating that the meta-analysis results were relatively stable.

In addition, publication bias was indicated among the studies of e-cigarette awareness in non-smokers because of the inconsistent results of the Egger’s test (p = 0.902) and the Begg’s test (p = 0.014). No publication bias was observed among the other studies.

A high degree of heterogeneity was observed in the pooled awareness rate, previous and current use rates, and healthier perception rate in both the total population and the subgroups (I^2^-values > 75% and p-values < 0.001).

## Discussion

The quality of studies included in our systematic review was generally high. The meta-analysis showed that a substantial number of adults were aware of e-cigarettes and had previously used them. Furthermore, previous users of e-cigarettes were more likely to be current smokers, and current users of e-cigarettes were more likely to be former/ever smokers. In addition, compared with other subgroups, the users of e-cigarettes tended to believe that e-cigarettes were less healthy than conventional tobacco. At the same time, the cumulative meta-analysis found that awareness of e-cigarettes was increasing and that the use and healthier perception of e-cigarettes remained relatively stable at higher levels during the last two years. These findings underscore the need to evaluate the potential long-term impact of e-cigarette use on consumer health, cessation, and nicotine addiction and to formulate a framework for e-cigarette regulation as soon as possible, which would enable the public to be fully informed and make good decisions about e-cigarettes.

### Awareness of e-cigarettes

The public’s awareness of e-cigarettes is generally high and is increasing. This study showed that current smokers were more likely to have heard of e-cigarettes than former smokers and non-smokers. Although few studies reported the sources of e-cigarettes awareness, the most common sources indicated by existing studies[13, 14] were the Internet, friends or personal contacts, and advertisements. For example, it was reported that during 2011–2012 in the US, e-cigarette advertising expenditures across media channels tripled from US$6.4 million in 2011 to US$18.3 million in 2012,[15] which was consistent with the increasing trend of awareness observed from 2012 to 2013 in this cumulative meta-analysis. Additionally, an internal debate over the safety and cessation properties of e-cigarettes in the public health community and the interest and concerns of the public are increasing rapidly [9], which might directly lead to increased awareness of e-cigarettes in general populations. However, we must stress that none of the studies reported the details of awareness by rating scores for knowledge through a series of questions instead of obtaining an affirmative or negative response to the simple question “Have you ever heard of electronic cigarettes, also called e-cigarettes?” As a result, we cannot conduct an in-depth exploration of the contents and level of knowledge about e-cigarettes among adults. Further study is needed to evaluate these awareness characteristics and their association with beliefs and behaviors.

### Previous and current use of e-cigarettes

This study showed that the pooled prevalence of former users of e-cigarettes was 16.8% (95% CI: 14.0–19.6%) and was most heavily concentrated among current smokers, at 27.2% (95% CI: 18.8–35.5%). The National Cancer Institute (2008) reported that tobacco advertising played an important role in consumers’ brand preferences, smoking initiation, and cigarette consumption. We speculate that the decision to use e-cigarettes is frequently driven and reinforced by how they are marketed. A content analysis reviewed 59 single-brand e-cigarettes retail websites in the US in 2012 and found that that the most popular claims were that the products were healthier (95%), cheaper (93%), and cleaner (95%) than regular cigarettes; could be smoked anywhere (88%); could be used to circumvent smoke-free policies (71%); did not produce secondhand smoke (SHS) (76%); and were modern (73%)[16].

Similarly, another content analysis reviewed a total of 18 websites of 12 e-cigarettes manufactures in China in 2013. It also found that the most frequent claims were health-related benefits (89%), followed by the claims of no SHS exposure (78%) and utility for smoking cessation (67%)[17]. Therefore, these advertising campaigns that claimed that e-cigarettes did more good than harm have inevitably caused to their high prevalence of use among the public, especially current smokers who may be more sensitive to tobacco advertising than other populations. Considering that there are no regulatory controls for the marketing of e-cigarettes and that few studies have investigated the safety, efficacy for harm reduction and cessation, and impact on public health, claims of health benefits, no SHS exposure, and value as smoking cessation aids should be prohibited by regulators until these issues have been adequately investigated.

The meta-analysis showed that current e-cigarettes users were likely to be former/ever smokers because former/ever smokers who had successfully quit smoking might have a stronger motivation and will-power and more willing to use tobacco substitutes such as e-cigarettes than current smokers. In contrast, because current smokers generally have greater nicotine dependence and addiction, e-cigarettes might not satisfy their taste of smoke and nicotine dose, which may have led them to turn to conventional tobacco after trying e-cigarettes. However, it remains uncertain whether e-cigarettes serve as a gateway to future tobacco use or lead to dual use of e-cigarettes and regular cigarettes due to limited prospective studies and inconsistent evidence for the alleviation of specific withdrawal symptoms [9]. The results of the meta-analysis are tentative concerning whether the high prevalence of e-cigarettes use occurs in other populations such as non-smokers. This issue needs to be investigated in a longer follow-up study.

### Healthier perception of e-cigarettes

This study found that more than half of the participants (52.6%, 95% CI: 42.5–62.6%) had the perception that e-cigarettes were healthier than traditional tobacco, which were also taken as common reasons for using e-cigarettes or a product that is healthier than cigarettes to quit smoking, as reported by many previous studies[18–22]. The health belief model (HBM), developed in the 1950s by psychologists from the US Public Health Service, holds that beliefs and attitudes can drive behaviors, including risky and health-protective behaviors. Based on this model, we speculate that the matching of beliefs with behaviors in this study might be due to the fact that e-cigarettes are advertised as a safer, more convenient, and socially acceptable alternative to smoking tobacco cigarettes.

However, a study of available toxicology data [23] indicates that very few commercially marketed e-cigarettes have undergone a thorough toxicology evaluation and standardized testing for evaluating e-cigarette toxicity across brands. In addition, a comparison to other tobacco products does not currently exist and publicly available high-quality scientific data on e-cigarettes is lack. Although e-cigarettes purportedly do not produce a combusted smoke, they deliver an aerosol containing nicotine and other tobacco-related compounds. However, knowledge is limited regarding the nicotine pharmacology and nicotine dependence of e-cigarettes and public health [24]. This knowledge is needed to understand the potential impact on individual users. Additionally, WHO is currently working with national regulatory bodies and toxicology experts to examine regularity options to understand more about the long-term impacts of e-cigarettes on health.

It is clear from the above discussion that there is still an unresolved tension between the harm-reduction goal of offering safer options to smokers and those of e-cigarettes makers being commercially viable and profitable. In the meantime, the present study found that the healthier perception has been relatively stable at a high level of 40% to 60% across the subgroups of different smoking status during the last few years. Thus, it is critical to develop appropriate health campaigns to inform e-cigarette consumers of the potential harms associated with e-cigarette use.

Notably, the present study found the lowest prevalence of healthier perception of e-cigarettes was among current e-cigarette users. A possible reason for this result is the large discrepancy in rates across the included studies, which ranged from 6% to 84%. The variations might be due to differences in the populations, survey time, and location. Although the sensitivity analysis showed that the removal of each study resulted in some change in the pooled estimates with overlapped 95% confidence intervals, from 26.1% (95% CI: 9.8–42.3%) to 46.2% (95% CI: -2.2–94.6%), the interpretation of the results should be cautious because of their large confidence intervals even the occurring of negative lower boundaries for 95% CIs.

### Strengths and limitations

This study first provided a quantitative synthesis about e-cigarette awareness, the prevalence of previous and current use, and perceived health risks so that the public and policy makers could be informed of the patterns of e-cigarettes use and healthier perceptions about this controversial product across populations and time. Using Preferred Reporting Items for Systematic Reviews and Meta-Analyses (PRISMA), we tried to improve the reporting (S1 Table). However, there were several limitations in this study. Firstly, the significant heterogeneity in this study cannot be ignored. To investigate possible sources of heterogeneity, we performed subgroup and sensitivity analyses, which provided an inadequate explanation for the heterogeneity findings. The high degree of heterogeneity observed may be due to the differences in time, place, methodologies of data collection, and surveyed populations. It is recommended that a standard methodology be proposed for sampling method and survey method and a periodic study at national and international levels should be carried out to provide a more comprehensive viewpoint.

Secondly, since we reviewed only articles written in English, this review may have missed some relevant articles. Thirdly, many of the studies included in the meta-analysis reported a high non-response rate, although the researchers had adjusted these rates based on the representative population. However, the non-response bias may be unavoidable. Finally, relying on self-report of the awareness of e-cigarettes, previous and current use, and healthier perception is likely to impact the sensitivity and specificity of the estimates.

In conclusion, the awareness of e-cigarettes has increased considerably in the past few years, and the use of e-cigarettes remained at a high level among adults during 2009–2014. Given the heterogeneity in this meta-analysis and the uncertain impact of e-cigarettes on public health, standardized survey methods at national and international levels, as well as continued surveillance of emerging utilization patterns of e-cigarettes are critical for public health policymaking.

## Supporting Information

S1 File Stata data files for meta-analysis. (RAR)

S1 Table PRISMA 2009 checklist. (DOC)
